# SURVEILLANCE OF CHILD DEVELOPMENT: AN ANALYSIS OF BRAZIL’S SITUATION

**DOI:** 10.1590/1984-0462/;2017;35;1;00009

**Published:** 2017-02-20

**Authors:** Maria de Fátima Costa Caminha, Suzana Lins da Silva, Marília de Carvalho Lima, Pedro Tadeu Álvares Costa Caminha de Azevedo, Maria Cristina dos Santos Figueira, Malaquias Batista

**Affiliations:** a Programa Nacional de Pós-Doutorado (PNPD) da Coordenação de Aperfeiçoamento de Pessoal de Nível Superior (CAPES) no Instituto de Medicina Integral Professor Fernando Figueira (IMIP), Recife, PE, Brasil.; b IMIP, Recife, PE, Brasil.; c Universidade Federal de Pernambuco (UFPE), Recife, PE, Brasil.; d Faculdade Pernambucana de Saúde (FPS), Recife, PE, Brasil.

**Keywords:** Child development, Primary health care, Comprehensive healthcare

## Abstract

**Objective:**

To describe Brazil’s historical background with regard to child development surveillance and perform a systematic review of studies published on surveillance records of child development within Child Health Handbooks.

**Data sources:**

A literature review was conducted in April of 2016 in the following electronic databases: Latin American and Caribbean Literature in Health Sciences (LILACS), the Scientific Electronic Library Online (SciELO), and the Medical Literature Analysis and Retrieval System Online (Medline). The search did not have any language or publication period restrictions, and included the bibliographic references of the selected articles. The keywords “child development and child health records,” and “child development and child health handbook” were applied. Articles were included that were original and that evaluated the use of child development surveillance tools in Brazil. Publications that were not original were excluded. The articles were selected first based on their title, then their abstracts, and finally a thorough reading.

**Data synthesis:**

The recommendation to support child development surveillance has been occurring since 1984. In 1995, developmental milestones were included in the Child’s Health Handbook, and in 2004 they became normative acts for surveillance, which should be carried out using this booklet. In the systematic review, six articles were selected in which the prevalence of child development surveillance recording ranged from 4.6 to 30.4%. This variation was due to different criteria and sample sizes as well as different methodologies employed to analyze the adequacy of filling out the handbook.

**Conclusions:**

Despite the fact that the Brazilian Ministry of Health formalized child development surveillance 32 years ago, the act of recording the surveillance in the Child Health Handbook is still deficient and irregular.

## RESUMO

**Objetivo:**

Descrever o caso do Brasil sob o aspecto de antecedentes históricos e realizar revisão sistemática de estudos publicados sobre registro da vigilância do desenvolvimento infantil mediante aplicação do Cartão ou Caderneta de Saúde da Criança.

**Fontes de dados:**

Fez-se busca da literatura em abril de 2016 nas bases eletrônicas: Literatura Latino-Americana e do Caribe em Ciências da Saúde (LILACS), Scientific Electronic Library Online (SciELO) e Medical Literature Analysis and Retrieval System Online (Medline), sem restrição do idioma nem período de publicação, e em referências bibliográficas dos artigos selecionados. Descritores utilizados: *desenvolvimento infantil e cartão da criança* e *desenvolvimento infantil e caderneta de saúde da criança*. Critério de inclusão: artigos originais que mensuravam, no Brasil, o uso do instrumento de acompanhamento do desenvolvimento infantil. Critério de exclusão: outra forma de publicação que não fosse artigo original. Os artigos foram selecionados pelos títulos, seguido dos resumos e de sua leitura na íntegra.

**Síntese dos dados:**

A recomendação em apoiar a vigilância do desenvolvimento infantil ocorre desde 1984. Em 1995, incluíram-se marcos do desenvolvimento no Cartão da Criança, tornando-se tais marcos, em 2004, ato normativo para registro da vigilância por meio desse instrumento. Na revisão sistemática foram selecionados seis artigos, nos quais verificou-se a prevalência de notificação da vigilância do desenvolvimento infantil de 4,6 a 30,4%. Essa variação deve-se a critérios e tamanhos amostrais diversos e a diferentes metodologias de análise sobre a adequação do preenchimento do cartão.

**Conclusões:**

Apesar de a formalização pelo Ministério da Saúde do Brasil da vigilância do desenvolvimento infantil ter ocorrido há 32 anos, quando avaliada pelo registro no Cartão ou Caderneta de Saúde da Criança, sua realização ainda é deficitária e irregular.

## INTRODUCTION

Established in the first half of the twentieth century as one of the conceptual and operational foundations of pediatric care, child development, in addition to somatic growth, represent some of the most important topics that define and qualify the active and continuous process of child health surveillance. In other words, in the way that growth and development are responses to standards or milestones hoped for with regard to people’s genetically programed potential, its careful evaluation allows for its accompaniment in a timely and relevant manner. This evaluation, upon completion, provides a sensitive parameter of the health/sickness process of an individual (or clinical) or collective (epidemiologic) level.^1^ ^,^ ^2^ ^,^ ^3^

Within two approaches, the historical principles of so-called social pediatrics, mainly represented by Robert Debret in France, David Morley in England, Julius Richmond in the United States, Frederico Gomez in México, Fernando Figueira, Martagão Gesteira, Martinho da Rocha, Gomes de Mattos, Pedro de Alcântara in Brazil, among others stand out.^4^ Such principles and practices are being updated and consolidated in the most recent consensus, for example, the recognizing and prioritizing of what’s called the “1,000 critical days,” understood as the nine months of fetal life and the first two years after birth, and which represents a period marked by vulnerability in terms of child survival and development^5^ in addition to the time for proper stimulation.^6^

Serving as a reference for politics and public health programs, two historical sequences exist. The first stage is characterized by high infant mortality due to the occurrence of deficiency diseases associated with infections. In this way, surveillance is valued more than growth as shown by graphs and anthropometric classifications.^7^ ^,^ ^8^ In the second stage, with material and social progress related to access and the resoluteness of health actions, a scenario is defined in which the developmental value is justified as an expected sequence in the transition process.^2^ ^,^ ^9^

There is no naturally occurring dividing line between the two scenarios. Therefore, these explanatory remarks lend themselves to illustrate the concept of typical epidemiological models that rarely exist in reality. In logical and conventional terms, prioritizing infant development would represent an advanced stage in the level of child health care. This is true for the context of most developed countries, which incorporate monitoring of development, standardized surveillance, triage and pertinent interventions. In the case of Brazil, analyzed as a special topic of this study, the justification for the systematic observation of child development should be configured regarding this new condition. This should have occurred starting in 2004, as an explicit recommendation for the application of children’s public policy.^10^

On an international level, as an innovative perspective on the idea of progressive application of new practices, environmental stimulation is already recommended for child development and should even start during fetal life. Many resources are used, like diversified food, a healthy mother transmitting the experience of flavor, a positive mood within the family, the importance of music, dialogue between mother and fetus carried out in a number of ways,^9^ ^,^ ^11^ ^,^ ^12^ reading out loud, highlighting skills they need to develop even before the conventional learning of reading and writing, which allows children to become more receptive to formal education at school, in addition to the experiences that strengthen the relationship between parents and children at a critical moment of development.^6^ ^,^ ^13^ ^,^ ^14^

In Italy, because of the project Nati per Leggere, which started in 1999, it is routine for the pediatrician, during primary health care, to advise the parents about the appropriate moment to read to their children. It demonstrates results from children at 5 years old and shows their breadth of vocabulary and comprehension skills-^14^ reading started in infancy equalizes the richness of vocabulary and the familiarity with school classes between the ages of 10 and 16 years old, independent of the economic condition of the family.^15^ Parental sensitivity should also be encouraged, in addition to the daily practice of healthy activities, the availability and use of toys, and other forms of precious stimulation, which favor brain formation and full development.^11^ ^,^ ^12^ ^,^ ^16^ Health professionals should provide the majority of this advice during home visits. It is known that families and communities value the advice of these professionals. A multidisciplinary team helps increase the family’s knowledge in their own bio psychosocial environment. It is the ideal moment to teach mothers and caregivers about the importance of child development.^17^ ^,^ ^18^

The objective of this article is to describe, contextualize, and establish some perspectives around the evolution of the concept of child development and its current and presumable breakdown at individual and collective level as a policy instrument and public health programs. Moreover, we intend to analyze the historic evolution of child development in Brazil and in agreement with an analytic point of view, understand the country’s situation, keeping in mind the perspective of evidence presented through the systematization of studies published in the last two decades.

### In the case of Brazil

An analysis of Brazil here is considered in two ways. The first constitutes an appreciation of the historical review that recognizes the growth and development proposition as a conceptual reference axis for the understanding and practice of monitoring the health/sickness condition of the child. The second way involves the search, selection, and systematization of published studies in Brazil about child development monitoring by means of Child Health Handbook records. In a historical aspect, the study has to do with an institutional itinerary, understood as policy positions, and programmatic actions of the Brazilian government regarding this issue.

Even though citations have been found in official documents, what really formalized a recommendation and a commitment on behalf of the Brazilian’s Health Ministry in supporting the surveillance of child development, was the creation of the Comprehensive Children’s Health Assistance Program (PAISC), officially started in 1984, 32 years ago.^19^ Nevertheless, the question of development was clearly undervalued in relation to other basic care needs treated in the referred to document as observed in concrete measure, like immunizations, the monitoring of development, breastfeeding, oral rehydration therapy (ORT), and other preventive and curative strategies applied to childhood illnesses.

High mortality rate in the 1980s focused the main objectives of the Brazilian government.^7^ ^,^ ^8^ Before then, there was no express recommendation to record the milestones of development in the Child Handbook, and consequently the demands that these record might indicate. From this perspective, the idea of child development only started to become a reference and was officially recommended regularly by the Health Ministry in 2004. The issue was newly repositioned.

In 2005, the Child Handbook, which was then converted into the Child Health Handbook (CSC)^20^ started to constitute the main instrument to accompany and register infant health in primary care. In the case of development, the child’s milestones should be evaluated and filled out at every doctor’s appointment from birth until 3 years of age, allowing for the identification of special needs that require timely and appropriate care. Additionally, these registrations need to be viewed in all other services and levels of attention, providing support for the basic and complementary health care of the child.^21^

Another factor to be considered is the bibliographic contributions directed toward the recovery of studies about the Brazilian experience before child development surveillance through a systematic revision of studies published in the past two decades. This revision, which involves various other approaches, and implies very different results, was also the purpose of Almeida et al.’s study.^22^ The study intended to “evaluate the use of a child health monitoring tool, emphasizing the variables of monitoring, growth, and development,” and including the trajectory of pregnancy and birth, and specifically focused on the task of updating the analytical contributions with regard to filling out the Child Handbook or the CSC in Brazil. The revision mentioned here analyzes the bibliographic contributions which concern the surveillance of child development in its dynamics and results. This explains the differences between the two evaluations.

### Data Source

For this revision, which serves as the second part of the analysis of Brazil, Almeida et al.’s study was used as a support, after the necessary adaptations objectives and methodology were made.^22^ The article here focuses specifically on the task of updating the descriptive and analytic contributions concerning the filling out of the Child Handbook or the CSC in Brazil with regard to the surveillance of “child development,” which officially became a priority starting in 2004.^10^

The treatment presented here is in agreement with the norms of the Preferred Reporting Items for Systematic Reviews and Meta-Analyses (PRISMA)^23^ A search was done in April of 2016, which did not have any language or publication period restrictions in the electronic databases LILACS, SciELO, and Medline and in the bibliographic references of the selected articles of interest. The following descriptors and keywords were used: “child development” and “child handbook” and “child development and child health handbook.” Articles that were included had their objectives defined, sample characteristics of participants, and texts published in indexed journals that showed, in Brazil, the use of the child development surveillance instrument, elaborated and distributed by the Health Ministry starting in 1995. It was then when 11 milestones in child development were established, with space graphs for recording the age with which they were achieved. Publications that were not original were excluded, for example, editorials, books, technical reports, reviews, completion of course work by undergraduate or graduate students with restricted viewing within their institution, as well as evaluations with qualitative approaches.

## DATA SYNTHESIS

In the database search, 54 articles were found: 39 in LILACS, 7 in SciELO, and 8 in Medline. Two more articles were found in consideration of the list of references, and, in one, the evaluation was performed through childcare mirror records of children in Basic Health Units. As such, 56 articles were identified initially. Later, 38 of them were excluded, of which 33 were excluded after reading the titles. In them, there was a discrepancy in relation to our objectives and criteria of inclusion; the other 5 were repetitions. In tracking, after reading the abstracts of the remaining 18 articles, 6 were excluded because they corresponded to articles that did not evaluate the filling out of the Child Handbook or the CSC. Of the 12 articles that remained for thorough reading, according to the rules of eligibility, six were excluded, because they did not have an evaluation that had to do with the filling out of the Child Handbook or the CSC in Brazil nor the surveillance of child development. Finally, six articles were chosen, because their abstracts related exclusively to the evaluation of the child development record. They are shown in Table 1.

**Table 1:** Final results of studies included in the systematic revision about child development surveillance in the Child Handbook or Child Health Handbook in Brazil starting in 2005.

Authors	Place and year	Age, statistic criteria, and sample sizes	Criteria for appropriate form filling	Attendance record (%)
Palombo et al.^24^	BHU in a municipality of São Paulo, SP, 2013	<3 years. Estimated 50% of children with inadequate food; 3,904 children recorded in BHU. Confidence level of 95% and an error of 5%, 350 necessary, 185 were analyzed.	Not explained	7.0
Abud e Gaíva^25^	Vaccination campaign in Cuiabá, MT, 2011	<1 year. Stratified random sample covering 60% of the units drawn in health regions. Of 63, 38 unites were randomly selected. It included all children attended during the day of the vaccination campaign. 929 children were analyzed.	≥2 items filled out according to the current age of the child	4.6
Ceia e Cesar^26^	BHU in Pelotas, RS, 2009	<1 year. Sample based on 4,000 live births in Pelotas in 2007. 90% prevalence of attendance in child care, a precision of +3 and including 350 children. A random drawing of the 50 BHU, selecting aprioristically half: 19 (of 37) in the urban area and seven (of 13) in rural areas. 365 mirror-chips were analyzed.	Not explained	6.0
Da Costa et al.^27^	Household in two municipalities in Piauí, 2008	<5 years. Appropriate form filling percentage of 22% of the handbook, 4% error, 95% confidence level, power of 80%, reason for no treatment: treated from 1:9 (income distribution), the outcome prevalence between untreated from 30% and risk ratio of 2.0. 263 necessary. 342 children were analyzed.	Regardless of whether it’s updated or not	30.4
Alves et al.^28^	Vaccination campaign in Belo Horizonte, MG, 2006	Seven to 16 months. Based on the number of live births in Belo Horizonte from May of 2005 to January of 2006: 22,311. 65% form filling frequency, error of 5%, 95% IC, sample of 344 children. Distributed among the nine health regions based on the proportion of live births. In each region, two BHU were randomly selected and estimated to have completed more than 200 vaccine doses in <1 year on the day of vaccination in June of 2005. Interviewed the odd numbers of eligible mothers in order of arrival in each of the 18 BHU. 355 children analyzed.	≥3 assessment records	18.9
Vieira et al.^29^	Vaccination campaign in Feira de Santana, BA, 2001	<1 year. Casual simple random sample of 62 units used for vaccination, 22 selected randomly. 2,191 children analyzed.	Notes corresponding to the child’s age	7.8

BHU: Basic Health Unit; IC: confidence interval.

According to the studies analyzed,^24^ ^,^ ^25^ ^,^ ^26^ ^,^ ^27^ ^,^ ^28^ ^,^ ^29^ the prevalence of child development recording in the CSC, with criteria and various sample sizes and methodologies analyzing the adequacy of filling in the variables, demonstrated a minimum value of 4.6% in Cuiabá, Mato Grosso (2011),^25^ and a maximum value of 30.4% in two places in Piauí in 2008.^27^ The last evaluation was performed in 2013,^24^ with a prevelance record of 7% in a municipality of São Paulo. It is worth noting that none of the studies mentioned in their methodologies that health professionals, in other words, had performed the child health surveillance evaluation through the Child Handbook or the CSC with notes during all child visits, according to age of the children. This shows a clear overestimation of these results, considering that ideally, a sequential and continuous record, covering each doctor’s appointment, as is recommending by the technical standard of the Ministry of Health would be completed.

## DISCUSSION

As a clinical and epidemiological concept, the definition of child development as a biological process, which is very characteristic of children, already expresses a practically 100 yearlong consensus. It is understandable both through observation and common sense that children should grow and flourish according to expected standards.

Nevertheless, what should be considered as an object of study is, effectively, how theses fundamental concepts, which are well accepted and recommended, are incorporated as individual care practices or as political and governmental support programs, revisiting, preliminarily, the innovative dimensions, and proposals so that the traditional concept of development is renewed in a way that assumes new roles, and, meanwhile, new demands in basic child health care.

The analyses presented here, which uses a recovered history about Brazil or which systematize the analytic studies of the selective bibliography, are consistent and conclusive, and recognize that child development with surveillance of the health/sickness process of Brazilian children is fundamentally neglected from central management to local assistance units.

Indeed, even considering the PAISC institution as a normative precursor for 32 years (1984-2016), historical documentation is very conclusive in making evident the gap between policy statements and related practical actions. As such, despite the graphic models that have already been available since 1995 for the registration of expected and achieved development milestones in the Child Handbook, child development surveillance still has not been taken on as a systematic and normative activity, in other words, as a recommendation from politics and public actions in health,^10^ which can explain the unexpressed percentages found in the selected studies. Even thought they were not included in the systematic revision because they were studies published in research reports and not in indexed magazines, two household surveys of the population base in the state of Pernambuco show that in 1997, the percentage of filling out of the child development surveillance was 1.1% and in 2006, 4.0%.^31^ This is therefore the item of recommendations that is usually not observed, considering the health professionals’ and the population’s lack of belief in the importance of this basic care.

Through the results of various studies, it is known that there are common difficulties in the filling out of the Card or the CSC,^32^ ^,^ ^33^ ^,^ ^34^ like in the illustrative case of a study with nurses and doctors from ESF in Belo Horizonte (MG), which tries to understand the lived experiences with the CSC, especially the difficulties of the surveillance process, an inadequate organization of the daily work of the teams, the mother’s lack of interest, and the lack of knowledge about the instrument.^32^ These restrictions were also found in Family Health Strategies in João Pessoa (PB) in a study with 45 nurses and 450 mothers with kids younger than two years old. The study is structured as an intervention, using a “before and after.” In the study, initially, interviews with the mothers about these practices and the completion of workshops with the professionals involved were evaluated, in addition to the knowledge practices of the nurses with respect to the surveillance of child development. A reevaluation of the results was completed after 4 months, when an increase in the implementation of surveillance of child development was observed.^33^ In a situation, diagnosis of the accompaniment of the growth and development of 1-year-old children, from the metropolitan region of Recife (PE) and the interior of the state of Pernambuco, in a sample of 816 evaluated children in 120 health units, it was found that 15.8% of the health units did not have the CSC and 75.4% did not have the development accompaniment norms,^34^ despite the fact that the childcare activities in basic care were attributed to overcrowding of nursing professionals in these units. However, the childcare service in primary care is commonly guided by complaints, in which the patient assumes a passive condition.^35^

The results of this revision do not differ much from the results found in medical practices in relation to the surveillance of child development in Brazil, just like in other countries. This is proved through a revision of Brazilian studies between 2000 and 2011, which show problems, from the schooling of the pediatrician to his or her clinical practice,^36^ which are similar problems to developed countries like the United States, where despite the use of a formal tool to evaluate child development during a medical practicum has doubled between 2002 and 2009, less than half of all pediatricians apply this tool in children under 36 months.^37^

It is estimated that, on a world level, 200 million children under 5 years of age stop fulfilling their full potential with regard to cognitive and socioemotional development, and countries from Sub-Saharan Africa have the highest percentage of disadvantaged children.^2^ In a cohort of births in New York City, United States, from 1994 to 2011, 45,709 (8.4%) children were identified with a delay in development.^38^ In Esmirnam, Turkey, a study involving 1,514 children between 3 and 60 months old, attended in 12 basic unites during approximately one year (2013-2014); the prevalence of delay was 6.4%.^39^

In Brazil, outside of the object of study mentioned and considered here (the registration of developmental milestones in the Child Handbook or CSC), it is interesting to notice some results that refer to their interpretation as an indicator of delay. Despite the limitations of the studies in relation to samples, age, diversity monitoring, and development screening tools, the prevalence of delay situations varies from 30 to 56% in cities in the states of Paraíba, Bahia, Minas Gerais, Goiás, and São Paulo,^24^ ^,^ ^40^ ^,^ ^41^ ^,^ ^42^ ^,^ ^43^ ^,^ ^44^ ^,^ ^45^expressing a worrying situation in that these frequencies are 6-20 times more elevated than the deficits appointed by anthropometric indicators of growth (weight/age, weight/height, and height/age). Taking into consideration that growth delay indicators demonstrate that the situation of nutritional deficit is believed to be practically resolved even in the poorest regions of the country,^46^ ^,^ ^47^ the idea stands that the moment of valuing child development, in a way that does not deface even more the registration of its surveillance, in agreement with the age appointed in the CSC, during a child’s well visit, characterizing and deepening an oversight that already is unacceptable.

It is noteworthy that, of the seven studies about the prevalence in development delay,^24^ ^,^ ^40^ ^,^ ^41^ ^,^ ^42^ ^,^ ^43^ ^,^ ^44^ ^,^ ^45^there was only one case of delay referral.^43^ Despite the knowledge of the potential of change inherent in neural plasticity,^9^ ^,^ ^48^ it provides warning for future problems with regard to children diagnosed with delay in their school age, such as restrictions on learning yield, low participation in the context of school activities, and significantly lower functional performance compared to children without delay history.^49^

Although there are limitations and lack of studies, the relaxed attitude generalized in relation toward child development surveillance in basic health attention becomes evident. It is possible to conclude that in Brazil, this care is the missing link in the chain of actions that should have been constituted for 32 years in the Comprehensive Health Care Program for women and children.^19^ In 2004, a commitment toward the surveillance of child development was reaffirmed, as required in the registration of the Child Card Handbook in basic health units,^10^ but even so, the progress has been insignificant. This limitation worsens upon being recognized and the emphasis with which it is prioritized on a world level, the importance of intensifying the care in what is called the “1,000 critical days” of the child.^5^ It is a matter that should be taken to national and international health forums, to executive and legislative powers and even to judiciaries as a human rights problem, even though we recognized that public opinion is not interested in the subject.
